# More than a Movement Disorder: Non-Motor Features and Future Directions in Dystonia Research

**DOI:** 10.3390/brainsci15121293

**Published:** 2025-11-29

**Authors:** Sanketh Rampes, Amit Batla

**Affiliations:** 1 Department of Neurology, Luton and Dunstable Hospital, Luton LU4 0DZ, UK; 2 Department of Clinical and Movement Neurosciences, University College of London Institute of Neurology, Queen Square, London WC1N 3BG, UK

**Keywords:** dystonia, non-motor symptoms, neuropsychiatric symptoms, cognition, sleep, quality of life

## Abstract

Background: Dystonia, the third most common movement disorder, is increasingly recognised as a network disorder with both motor and non-motor symptoms. Non-motor symptoms have been shown to be key determinants of quality of life in dystonia and include anxiety, depression, sleep disturbance, cognitive dysfunction and fatigue. Results: Emerging data suggests that dysfunction within cortico-striato-thalamo-cerebello-cortico circuits underpins both motor and non-motor symptoms. Genetic studies have highlighted shared gene clusters involved in synaptic function that are associated with both dystonia and psychiatric disorders. Neuroimaging studies reveal microstructural and functional alterations in patients with dystonia that correlate with non-motor symptoms. Discussion: Current research into both the pathophysiology and treatment of non-motor symptoms remains limited, heterogeneous and based on small sample sizes, which restricts the strength of the conclusions that can be drawn. Evidence for targeted therapies for non-motor symptoms is scarce. Conclusions: A greater understanding of the overlap between neural pathways underpinning motor and non-motor symptoms may provide a foundation for the development of novel pharmacological and non-pharmacological therapies. As understanding advances, treatment strategies will likely adopt a holistic model that integrates pharmacological options with non-pharmacological measures, including multi-disciplinary rehabilitation and supportive therapies.

## 1. Introduction

Dystonia is the third most prevalent movement disorder [1]. Epidemiological data from recent studies estimate the prevalence of isolated dystonia to be between 30.9 and 52.7/100,000 [2,3]. Dystonic movement and postures can often be disabling and are initiated or worsened by voluntary action and frequently associated with overflow movements [4].

Although non-motor symptoms are not included in the current dystonia classification system [4], there is a growing body of evidence that demonstrates that anxiety, depression, pain and sleep disorders significantly contribute to morbidity and quality of life in individuals with movement disorders [5,6]. Non-motor manifestations encompass depression, anxiety, pain, sleep, cognition and fatigue. In a similar way to Parkinson’s disease, non-motor symptoms in dystonia have been shown to occasionally predate motor symptoms and be a distinct feature of the condition, unrelated to the degree of motor impairment. Although they have been shown to occur independently of motor symptoms, they commonly interact, making it challenging to investigate each symptom individually [7].

In this review, we integrate current evidence on the pathophysiology, epidemiology and treatment approaches for non-motor symptoms in dystonia. We highlight shared neural pathways underlying motor and non-motor symptoms and present a case for integration of non-motor symptoms into research and day-to-day clinical practice, which may enable the development of target therapies for patients.

## 2. Pathophysiological Mechanisms of Non-Motor Symptoms

**Figure 1:**
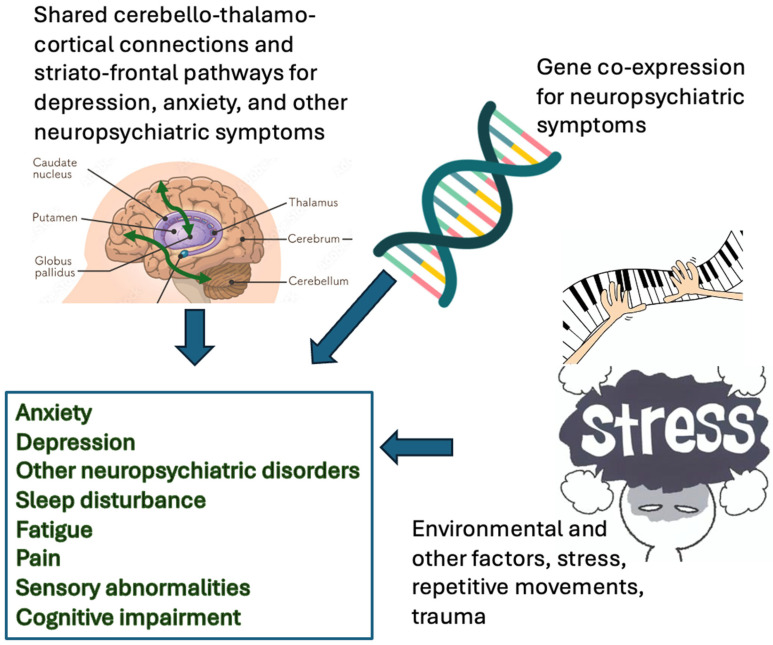
Overview of the mechanisms contributing to non-motor symptoms in dystonia. The figure highlights the convergence of shared cerebello-thalamo-cortical and striato-frontal pathways relevant to depression and anxiety, genetic co-expression of neuropsychiatric-associated genes, and environmental stressors (including stress, trauma and repetitive movements) in driving non-motor manifestations.

Movement is a complex process that involves planning, coordination and execution of movement; all of which are carried out by different regions of the brain. Dystonia is accepted to be the result of a dysfunctional network involving the cortex, thalamus, basal ganglia, brainstem and cerebellum [8]. The cerebellum has been identified as a key structure in the network of dystonia, contributing not only to motor control but also to non-motor features through aberrant cerebello-thalamo-cortical connectivity [9,10,11]. The body of evidence to date supports the idea that rather than arising from a single area within the brain, it involves a dysfunctional network involving the cortico-cerebello-thalamo-cortical loops and cortico-striato-thalamo-cortical [8,12] (Figure 1).

Functional brain imaging has shown that compared to control subjects, patients with dystonia demonstrate distinct temporal and spatial patterns of neural activity across the motor cortex, cerebellum, globus pallidus internus (GPi) and putamen [13]. Disruption of these same circuits has also been implicated in neuropsychiatric symptoms [14]. White matter microstructural changes have also been shown utilising ultra-strong diffusion gradient MRI in patients with cervical dystonia [15]. These white matter microstructural changes were found to correlate with non-motor symptoms. Psychiatric symptoms, specifically obsessive compulsive symptoms, were found to correlate with left anterior thalamic radiation *p*_2_ (r = 0.92, *p* < 0.001); sleep quality, as measured by Sleep Disorders Questionnaire Score, was found to correlate with left anterior thalamic radiation ODI (r = −0.84, *p* < 0.001); pain was found to correlate with the left anterior thalamic radiation ODI (r = −0.89, *p* < 0.001); and cognitive functioning was found to correlate with paired associate learning task p2 (r = 0.94, *p* < 0.001) [15].

An unbiased systems-biology approach was employed to examine the relationship between genes associated with dystonia and neuropsychiatric disorders. Gene network analysis revealed that gene clusters enriched in dystonia confer increased genetic risk of psychiatric disorders, particularly within the white matter modules, frontal cortex and putamen [16]. Enrichment of these gene clusters for synaptic function-related genes supports the idea that psychiatric manifestations in dystonia are likely intrinsic to its pathophysiology [16]. Several molecular pathways have been identified in connection with dystonia-causing genetic variants. These molecular pathways include: gene transcription during neurodevelopment (e.g., KMT2B, THAP1), striatal dopamine signalling (e.g., GNAL), calcium homeostasis (e.g., HPCA, ANO3), autophagy (e.g., VPS16), endoplasmic reticulum stress response (e.g., TOR1A, PRKRA, EIF2AK2) and others [8]. Some of these overlap with psychiatric disorders, particularly this link with genetic expression is supported by evidence from DYT-11 associated with epsilon-sarcoglycan gene (SGCE) mutations. This condition is associated with specific psychiatric disorders, most commonly OCD, anxiety-related disorders, and alcohol dependence [17]. These possibly suggest a potential pleiotropic function for SGCE within the central nervous system that might predispose to specific neuropsychiatric manifestations.

Our understanding of the pathophysiology of non-motor symptoms in dystonia is currently limited by the paucity of research available. The studies to date mostly consist of small sample sizes and vary in methodology, limiting the generalisability of the findings. Future studies, including genetic and imaging studies, should aim to link both motor and non-motor symptoms and identify shared neural pathways.

## 3. Neuropsychiatric Symptoms in Dystonia

Patients with dystonia, including those whose dystonia has an underlying genetic aetiology, have an increased burden of psychiatric symptoms, in particular anxiety and depression, which are also the most significant determinants of disability and decreased quality of life (Table 1) [18,19,20,21]. A recent large population study from Sweden has shown that individuals with dystonia when compared to those without, were more likely to have a diagnosis of anxiety disorder (adjusted odds ratio = 2.13, 95% confidence interval 1.90–2.39), depression (adjusted odds ratio = 2.00, 95% confidence interval 1.77–2.26) and suicide attempts or death by suicide (adjusted odds ratio 1.90, 95% confidence interval 1.50–2.17) [21]. These findings extended to siblings, who exhibited increased likelihood of psychiatric disorders and suicide attempts or deaths relative to siblings of people without dystonia [21]. Another large population study additionally looked at the temporal relationship between psychiatric symptoms and the diagnosis of dystonia, and found that the diagnosis of psychiatric disorders predominantly predated the diagnosis of dystonia (incidence rate ratio [IRR] = 1.98, 95% confidence interval 1.9–2.1), with this relationship most prominent for anxiety disorders (IRR = 12.4, 95% confidence interval 11.9–13.1) [20]. This supports evidence from previous studies identifying pre-motor onset of psychiatric symptoms [18].

The prevalence of psychiatric symptoms across dystonia subtypes varies. A large international cohort study of 478 patients found that levels of anxiety varied by dystonia onset site, with higher rates seen in patients with cervical and laryngeal onset [22]. This was in contrast to depressive symptoms, which were not found to significantly vary between onset sites when adjusted for pain and motor severity [22]. A large systematic review and meta-analysis of 54 studies and 12,635 patients identified a pooled prevalence of depressive symptoms/disorders of 29.2% for cranial dystonia and 31.5% for cervical dystonia [19]. The same group conducted another meta-analysis and systematic review involving 34 studies, which found clinically meaningful anxiety symptoms and anxiety-related disorders in 40% (95% confidence intervals 20 to 60%) in cervical dystonia and 25% (95% confidence intervals 21 to 30%) in cranial dystonia [23]. In cervical dystonia, social anxiety has been reported at rates up to 57% [22]. Social anxiety has links to the individual’s body concept, highlighting the complex interplay between social stigma, social anxiety and body concept [24].

In monogenic dystonia, both non-manifesting and manifesting TOR1A mutation carriers exhibit an increased risk of major depressive disorder in comparison to individuals without the mutation [25]. A recent systematic review found that psychiatric comorbidities occur more frequently in manifesting DYT-SGCE mutation carriers compared to non-manifesting carriers or those without the mutation [26]. There is limited evidence regarding the relationship between anxiety disorders and monogenic dystonia [26].

## 4. Cognitive Impairment in Dystonia

Cognition in dystonia has mainly been focused on adult-onset forms, in particular cervical dystonia, and studies have thus far yielded mixed results, which means that there are no clear signatures for cognitive deficits in dystonia. This has also been confounded by the high variability in the use of tools to assess cognitive function, lack of standardisation in research methodology and use of small cohorts without adequately matched control populations [27,28,29]. Future research should aim to include all dystonia subtypes to look for between-group differences.

A study of 25 primary dystonia patients found frequent cognitive impairment compared to demographically matched controls. Individuals with dystonia performed worse on measures of memory, conceptualisation, attention and global cognitive function (*p* < 0.001 for all measures) [28]. In the same study, 80% of dystonia patients demonstrated deficits on at least one neurocognitive assessment, with more than 60% exhibiting impairments across three or more measures [28]. Other studies have found more limited evidence of cognitive impairment; Maggi et al. found that patients with focal dystonia, when examined across multiple cognitive domains, only exhibited differences in prospective memory, which was poorer when compared to unaffected controls on tests involving time-based (*p* < 0.001) and recognition components (*p* = 0.013) [30].

Within the domain of cognition, research has primarily focused on three key areas: dual tasking, social cognition and auditory processing [31]. There is evidence for alterations in the processing of sensory input in dysfunction, which is thought to result from a reduction in lateral inhibition within the brain’s sensory regions [31,32]. Individuals with cervical dystonia have demonstrated decreased selective attention (*p* = 0.001), divided attention (*p* < 0.001) and visual processing speed (*p* < 0.001), suggestive of impaired visual attention and functional vision [33]. Another study of cervical dystonia patients revealed that those patients with torticollis showed a greater leftward deviation than the control group [34], suggesting possible impaired spatial attention. Patients with blepharospasm exhibited impairment in visuospatial working memory, complex movement planning and sustained attention [35]. The cognitive impairments were found to be independent of depression, anxiety and premorbid intelligence, supporting broad cortical involvement in dystonia and abnormal connectivity within fronto-striatal networks [35]. Social cognition describes the cognitive mechanisms involved in understanding and reacting to the emotions, intentions and actions of others. Studies of patients with cervical dystonia have shown impaired basic social cognition (*p* = 0.007), recognition (*p* = 0.006) and delayed recall (*p* < 0.001) when compared to healthy matched controls [36]. Other studies have also shown worse performance on the identification of emotional prosody and naming affect [37,38]. A possible explanation is localised disruption within the collicular-pulvinar-amygdala pathway [36]. Dual tasking has also been suggested as a cognitive domain affected in dystonia [31]. Patients with adult-onset focal dystonia show deficits in postural stability compared with controls that worsen under more demanding cognitive tasks (*p* < 0.0001) [39], along with slower gait, increased dual task cost and stance time [40].

## 5. Sleep Disorders and Fatigue

Early nocturnal polygraphic studies revealed reduced sleep efficiency, reductions in REM sleep and increased awakenings in blepharospasm, cranial and oromandibular dystonia [14,41,42]. Between 40% and 70% patients with dystonia experience sleep disturbance, most commonly abnormal nocturnal movements and insomnia [43]. The underlying causes of sleep abnormalities in dystonia, similar to other non-motor symptoms, may be intrinsic to the disorder or secondary to factors such as pain or pharmacological treatments. A systematic review indicated that sleep disturbances are reported by over 50% of patients with focal dystonia, although excessive daytime sleepiness is rare [44]. Sleep in a dystonia cohort was examined in a UK Biobank study through objective accelerometer data and subjective sleep questionnaires, and compared to findings in a control population. Patients with dystonia exhibited impaired self-reported sleep, and accelerometer data highlighted delayed sleep onset, circadian rhythm changes and decreased time in bed [45].

It is important to acknowledge the interplay between psychiatric comorbidities and sleep disturbances, and indeed, most sleep studies have examined for any possible interaction. Evidence shows that sleep disturbances are related to depressive symptoms (*p* < 0.001) and the presence of restless leg syndrome (*p* < 0.01); however, sleep disturbance was not correlated with the degree of motor symptom severity [46]. This is supported by a cohort study of Chinese adult-onset focal dystonia patients that showed no association between motor severity and non-motor symptoms [47]. Indeed, in patients with cervical dystonia, activity over cervical muscles reached significantly reduced levels in both REM and non-REM sleep when compared to controls [42].

There is scope for further research into how fatigue and dystonia interrelate. In a Botulinum Toxin clinic cohort of dystonia patients, 43% were found to have moderate to severe fatigue [48]. Fatigue has been shown to adversely affect quality of life, even when adjusted for concomitant depression [48]. Another study in patients with cervical dystonia found that fatigue was increased independently of psychiatric comorbidity (*p* < 0.01) and independently of motor severity [49]. Conversely, a more recent study of focal and segmental dystonia found that depressive symptoms predominantly predicted fatigue across general, mental and physical domains [50].

## 6. Pain and Sensory Symptoms

Pain is among the most commonly reported and disabling symptoms in dystonia, affecting an estimated 67 to 75% patients with cervical dystonia [51,52]. In cervical dystonia, the pain primarily involves the neck and shoulders and may spread to the side of the head deviation or the ipsilateral upper limb. Additionally, 10 to 20% of patients report a headache [53].

There is, in cervical dystonia, not a consistent correlation between pain and motor severity or posture. Even for patients with equivalent motor severity, the experience of pain can differ, with patients reporting different levels of pain [54]. This effect is additionally seen in the treatment of dystonia with Botulinum Toxin, where the impact on contractions and postures is sometimes non-congruent with pain [55,56]. This observation is supported by deep brain stimulation (DBS) studies, which demonstrate different time-scales for the improvement of posture deficits and pain [57]. Clinical observations indicate that the site of pain in dystonia does not always correspond directly to the affected muscles [54].

There is evidence suggesting that patients with dystonia have a reduced pain threshold [54]. In a study of nine patients, it was found that patients with cervical dystonia had a pain threshold roughly half that observed in age and gender matched individuals [58]. Another study in patients with focal hand dystonia found alterations in sensitivity and a great subjective sensation of pain, supporting decreased pain thresholds in dystonia [59]. The literature on chronic pain implicates dysregulation in descending pain modulation, and disruption in these modulatory circuits may contribute to chronic pain. In patients with cervical dystonia, impaired conditioned pain modulation has been observed, indicating dysfunction in descending pain inhibition [60].

Alterations in the somatosensory system are likely also implicated in the pathophysiology of pain in dystonia [61]. Alterations in the somatosensory system are well documented within dystonia, with abnormal mapping of dystonic body parts within the primary somatosensory cortex (S1), differences in excitability on neurophysiological studies and with movement-related changes in S1 cortical activity [62]. The involvement of somatosensory processing is also supported by the work of Pelosin and colleagues, who found improvements in pain in patients with cervical dystonia using sensory modulation techniques such as kinesiotaping [63]. Patients often use manoeuvres to help with dystonia termed sensory trick (or geste antagoniste), which underscores the contribution of sensory pathways to dystonia [64]. Impairments in sensory processing and in spatial and temporal discrimination of tactile input have been reported in dystonia [65,66].

## 7. Quality of Life and Functional Impact

Patients with any form of dystonia have been shown to have significantly impaired quality of life compared to healthy controls without dystonia [67]. Multiple factors contribute to this, including social and demographic factors, non-motor symptoms and social stigma [67]. Most research into quality of life in dystonia to date has focused on cervical dystonia and blepharospasm, reflecting the higher prevalence of these subtypes. Studies have shown that patients with generalised dystonia experience worse quality of life when compared with focal dystonia [5]. Other factors that were identified to correlate with worse quality of life included being unemployed, being divorced and being of younger age [5].

In dystonia, depression and anxiety are the most significant determinants of quality of life [67,68]. In cervical dystonia, altered body image and reduced self-esteem resulting from disfigurement are shown to be key contributors to depression, as evidenced by self-assessed disfigurement being identified as the key determinant of depression [69]. Studies have shown that successful treatment with Botulinum Toxin of head deviation, while effective for depression, has little impact on the patient’s negative body image [70], suggesting that interventions to improve quality of life should also be targeted at improving the body concept [5]. Overall, there is strong evidence that non-motor symptoms are strongly associated with poor quality of life and that investigating and treating these ought to be a standard component of clinical practice [71].

Ben-Shlomo et al. showed that individuals with increased duration of disease had an increased quality of life, possibly as they had longer to develop coping mechanisms [68], which might include personalised sensory tricks (for example, a scarf or a corset) and mechanical mechanisms (for example, ptosis crutches) [72]. They identified that poor social support and self-depreciation tendencies predicted worse mental health outcomes [68]. These findings are similar to comparable other chronic diseases and suggest that increased focus should be put on treating anxiety and depression and enhancing social support.

**Table 1:** Key studies referenced on non-motor symptoms.

Author	Year	Country	Sample Size	Key Findings
Neuropsychiatric symptoms				
Fabbrini et al. [18]	2010	Italy	*n* = 89, HC = 62	High frequency of depressive disorders in dystonia patients compared to controls.
Medina Escobar et al. [19]	2021	Multicentre (systematic review and meta-analysis)	*n* = 12,635	Pooled prevalence of depressive symptoms/disorders is 31.5% cervical dystonia and 29.2% cranial dystonia.
Bailey et al. [20]	2022	UK	*n* = 52,589, HC = 216,754	High rates of psychiatric diagnoses and psychiatric medication prescriptions in dystonia patients compared to controls.
Martino et al. [21]	2020	Sweden	*n* = 2958, HC = 6,841,937	Dystonia patients and their siblings had higher rates of depression, anxiety disorders or suicide attempts compared to controls.
Berman et al. [22]	2017	International multicentre	*n* = 478	High rates of anxiety and depression amongst all dystonia patients. Anxiety highest in cervical and laryngeal subgroups.
Medina Escobar et al. [23]	2021	Multicentre (systematic review and meta-analysis)	*n* = 6919	Pooled prevalence of clinically relevant anxiety symptoms and anxiety disorders was 40% for cervical dystonia and 25% for cranial dystonia.
Heiman et al. [25]	2004	USA	*n* = 96 (manifesting carriers), *n* = 60 (non-manifesting carriers), *n* = 65 (non-carriers)	Manifesting carriers of DYT1 mutation and non-manifesting carriers had increased risk of major depressive disorder when compared to non-carriers.
Lane et al. [26]	2021	UK	*n* = 544 (dystonia patients), *n* = 263 (control subjects with medical comorbidity), HC = 115	Study subjects with DYT-SGCE appeared at higher risk of psychiatric comorbidity than controls.
Cognitive symptoms				
Niccolai et al. [28]	2020	USA	*n* = 25, HC = 25	Dystonia patients performed worse on measures of global cognitive function, attention, memory and conceptualisation compared to controls.
Maggi et al. [30]	2019	Italy	*n* = 53, HC = 30	Selective deficit in prospective memory in focal dystonia patients.
Bastos et al. [33]	2021	Brazil	*n* = 50, HC = 50	Impaired visual processing speed, divided attention and selective attention in cervical dystonia patients compared with controls.
Chillemi et al. [34]	2017	Italy	*n* = 23, HC = 12	Biased spatial attention in patients with cervical dystonia compared to controls.
Alemán et al. [35]	2009	Argentina	*n* = 20, HC = 17	Impairment in visuospatial working memory, complex moving planning and sustained attention was found in patients with blepharospasm compared to controls.
Burke et al. [36]	2020	Ireland	*n* = 46, HC = 46	Patients with cervical dystonia were found to have impaired basic social cognition, recognition and delayed recall when compared to controls.
Rinnerthaler et al. [37]	2005	Austria	*n* = 32, HC = 32	Patients with dystonia showed isolated deficits in the recognition of disgust compared to controls.
Nikolova et al. [38]	2011	Germany	*n* = 30, HC = 30	Compared to controls, dystonia patients showed impaired processing of emotional prosody.
Baione et al. [39]	2021	Italy	*n* = 22, HC = 19	Patients with cervical dystonia showed impaired postural control compared to controls.
Crisafulli et al. [40]	2021	Italy	*n* = 17, HC = 19	Patients with cervical dystonia showed slower gait speed, longer stance time and increased dual-task cost when compared to controls.
Sleep				
Bailey et al. [43]	2023	UK	*n* = 241, HC = 964	Accelerometry measurements revealed later sleep times, reduced time in bed and shifts in circadian rhythms in dystonia patients compared with controls.
Paus et al. [46]	2011	Germany	*n* = 221, HC = 93	Impaired sleep quality was found in 44% patients with cervical dystonia, 46% patients with blepharospasm and 20% controls.
Yang et al. [47]	2016	China	*n* = 120, HC = 60	Dystonia patients showed impaired sleep quality when compared with controls.
Wagle Shukla et al. [48]	2015	USA	*n* = 91	43% of dystonia patients had moderate to severe fatigue, which negatively impacted quality of life even when adjusted for depression.
Smit et al. [49]	2016	The Netherlands	*n* = 44, HC = 43	In patients with cervical dystonia, fatigue was found to be independent of psychiatric comorbidity and a significant influence on quality of life.
Tomic et al. [50]	2022	Croatia	*n* = 60	67% of dystonia patients reported fatigue.
Pain				
Kutvonen et al. [51]	1997	Finland	*n* = 39, HC = 18	Two-thirds of patients with cervical dystonia reported continuous or intermittent recurrent pain.
Chan et al. [52]	1991	USA	*n* = 266	75% patients with cervical dystonia reported pain.
Quagliato et al. [55]	2010	Brazil	*n* = 24	Botulinum Toxin therapy was associated with improvements in pain in patients with cervical dystonia.
Camargo et al. [56]	2011	Brazil	*n* = 28	Botulinum Toxin is an effective therapy for pain in patients with cervical dystonia.
Kulisevsky et al. [57]	2000	Spain	*n* = 2	Case reports showing temporal dissociation between pain and motor improvements.
Lobbezoo et al. [58]	1996	Canada	*n* = 9, HC = 5	Reduced pain-pressure thresholds in patients with cervical dystonia compared to controls.
Pérez-de-Heredia-Torres et al. [59]	2022	Spain	*n* = 12, HC = 12	Patients with hand dystonia had greater subjective pain compared to controls.

*n* = number of patients; HC = healthy controls.

## 8. Therapeutic Approaches Targeting Non-Motor Symptoms

Little research is available on the treatment of non-motor symptoms in dystonia. This is likely because further research is required to assess, characterise and define non-motor symptoms to allow for the design of therapeutic trials. It is likely that we will see increasing numbers of studies investigating the treatment of non-motor symptoms in the coming decade.

Pharmacological treatments for non-motor symptoms remain largely unexplored. A randomised controlled trial (RCT) conducted over six weeks in individuals with cervical dystonia showed that escitalopram had no measurable effect on mood or psychiatric symptoms when compared to placebo [73]. In another RCT investigating escitalopram for the management of dystonic jerks, escitalopram was found to have no significant effect on quality of life or psychiatric symptoms relative to placebo [74]. Epidemiological studies have shown that roughly half of dystonia patients receive at least one prescription for an antidepressant or anxiolytic medication [20]. It would be informative to conduct retrospective studies evaluating for any class effects of different antidepressant medications on dystonia, in addition to well-designed randomised controlled trials.

Botulinum Toxin (BoNT) treatment has been demonstrated to alleviate pain in both oromandibular and cervical dystonia [7,75,76]. The impact of BoNT on sleep is inconsistent, with some studies showing benefit [76] whilst others showing no benefit [75]. Changes in motor severity often did not correlate with non-motor symptoms, including sleep, pain and mood, suggesting a direct effect of BoNT on these symptoms [7].

Multiple studies have assessed the impact of DBS on motor and non-motor symptoms in dystonia. The results of the studies are inconsistent, with some showing improvements in psychiatric or cognitive symptoms and others showing no difference [7,31]. Moriarty et al. showed that in patients with cervical dystonia, 24 months post-DBS, there was no significant difference in depressive symptoms (*p* = 0.5) and anxiety symptoms (*p* = 0.2919) when compared with the pre-morbid state [77]. Another study on seven patients with myoclonus dystonia also failed to show any improvement in depressive symptoms 6 months following DBS [78]. Studies on cognitive function largely show no change with DBS; however, one study showed modest improvements in phonemic verbal fluency (*p* < 0.05) at 12 months post-DBS [79] and demonstrated modest gains in executive function, which the authors suggested could be attributed to either surgical intervention or a reduction in anticholinergic medication use [80]. DBS does not appear to have a significant influence on sleep quality [81,82].

Cognitive-behavioural therapy (CBT) has been assessed in a small feasibility trial, suggesting that it may be of benefit for psychiatric symptoms in patients with dystonia. The feasibility trial investigated an 8-week internet-based CBT programme in 10 patients, and although there were no significant between-groups differences, anxiety and depression symptoms showed a trend towards improvement at the end of the three-month assessment period [83]. Patient engagement and satisfaction were also largely positive, providing further support for future larger-scale trials into CBT in dystonia.

Rehabilitation approaches, including physical therapy focusing on sensory-motor integration and occupational therapy to help with activities of daily living, may play a role in the holistic management of dystonia [84,85]. The diverse treatment approaches of rehabilitation have hampered efforts to determine its efficacy for motor symptoms, with the overall quality ranging from low to very low based on the GRADE system [84]. Few studies have looked at rehabilitation approaches specifically for non-motor symptoms. A randomised controlled trial evaluating physical therapy regimen on disability in cervical dystonia found significant benefits on motor and non-motor symptoms, namely improvements in severity of anxiety (*p* = 0.039) and depression (*p* = 0.003) [86]. There are some ongoing trials investigating the effect of exercise due to completed in the next few years [87].

Overall, the evidence base for treatment of non-motor symptoms remains highly limited. There is a paucity of research, and available research remains highly heterogeneous, comprising small sample sizes, and few are randomised controlled trials. As shown in Table 2, only two-thirds evaluate non-motor symptoms as a primary endpoint, further limiting the conclusions that can be drawn.

## 9. Future Directions and Research Gaps

The advance of research into dystonia has been highly productive over the past decades with respect to pathophysiology and treatment of motor symptoms. Although there has been significant advancement in the characterisation and epidemiology of non-motor symptoms, largely within focal dystonia, the pathophysiology of non-motor symptoms and treatment remains largely underexplored.

The shift towards dystonia as a network disorder has been largely welcomed. We propose that further research should be undertaken to integrate non-motor and motor symptoms into the same model. Research has shown that cortico-striatal-thamalo-cortical circuits and the cerebellum may explain the underlying shared pathophysiology of motor and non-motor symptoms in dystonia [11,14,88]. There has been seminal work on functional brain imaging in dystonia, which has revealed microstructural brain changes significantly associated with non-motor symptoms [15] and research that has worked at a systems biology level to identify shared genetic networks involved in dystonia and psychiatric disorders [16]. Further research using multimodal brain imaging combined with molecular biomarkers should aim to link motor and non-motor symptoms. This could lead to a better understanding of the overlap in the molecular and structural links between motor and non-motor symptoms in dystonia.

Overall, studies on non-motor symptoms are highly heterogeneous, reflective of the variable study design and quality. There is a need to standardise the assessment of non-motor symptoms in patients with dystonia to allow for better characterisation and monitoring of symptoms. Sleep disorders and fatigue remain less consistently studied compared to psychiatric symptoms and pain within dystonia. A single questionnaire for the assessment of non-motor symptoms has been developed and validated in a cohort of patients with cervical dystonia [89]. However, the questionnaire lacks granularity, combining motor and non-motor symptoms without assessment of severity or impact on daily function [27]. It is conceivable that this questionnaire could be expanded to include more detail to accurately capture all non-motor symptoms and their functional impact.

Research into treatment approaches for non-motor symptoms in dystonia remains highly limited. Further research should aim to study different therapies for the individual non-motor symptoms in dystonia, based on the preliminary trials that have been conducted to date. Further understanding of the pathophysiology of non-motor symptoms in dystonia will also allow for the development of targeted therapies within this cohort.

As research advances and the evidence base for treatment of non-motor symptoms in dystonia emerges, it will be necessary to develop better clinical pathways for dystonia. Most patients with dystonia see clinicians in a Botulinum Toxin injection clinic with inadequate time to fully assess non-motor symptoms and implement holistic person-centred care. There is a clear argument presented with evidence that supports that the clinical pathway in dystonia should allow more comprehensive follow-ups and address all associated non-motor symptoms. It may be worth learning from the similar practices in other complex neurological conditions like Parkinson’s and Multiple Sclerosis, where nurse specialists and community engagement are now standard practice [90].

## 10. Conclusions

There is now an improved understanding of the pathophysiological underpinnings of non-motor symptoms, which opens avenues for treatment with pharmacological and non-pharmacological treatments.

Non-motor symptoms, including anxiety, depression, sleep disturbance, cognitive dysfunction and fatigue, have significant impacts on quality of life in dystonia. Unfortunately, these remain under-recognised, underdiagnosed and undertreated in routine clinical practice. Whether the non-motor symptoms are secondary consequences to motor dysfunction, an associated burden or an integral component of the biological processes that cause dystonia, is difficult to conclude based on current evidence. There is, however, a clear shift towards the latter hypothesis with evidence emerging from molecular, genetic and functional brain imaging studies.

Future studies should include both mechanistic studies to identify possible treatment avenues and well-conducted trials evaluating treatments using non-motor symptoms as primary endpoints. Greater integration of non-motor symptoms into day-to-day clinical practice will enable more refined phenotyping and support the development of personalised treatment approaches.

## Figures and Tables

**Table 2:** Key studies evaluating treatments for non-motor symptoms.

Author	Year	Country	Sample Size	Study Design	Intervention	Key Findings
Treatment studies						
Zoons et al. [73]	2020	The Netherlands	*n* = 18 (10 intervention, 8 placebo)	Double-blind RCT	Escitalopram vs. placebo	No measurable effect on mood or psychiatric symptoms during 6-week period.
Zoons et al. [74]	2018	The Netherlands	*n* = 53	Double-blind randomised crossover trial	Escitalopram vs. placebo	No significant difference in psychiatric symptoms between groups.
Costanzo et al. [75]	2021	Italy	*n* = 45	Prospective	BoNT	In cervical dystonia patients, BoNT resulted in improvements in psychiatric symptoms, pain and disability.
Elshebawy et al. [76]	2025	Egypt	*n* = 40	Prospective	BoNT	In cervical dystonia and hemifacial spasm patients, BoNT was associated with improvements in psychiatric symptoms, pain and sleep quality.
Moriarty et al. [77]	2022	Ireland	*n* = 53	Prospective	BoNT	No significant difference in psychiatric symptoms, quality of life or pain in cervical dystonia patients treated with BoNT over a 24-month period.
Krause et al. [78]	2021	Germany	*n* = 7	Retrospective	DBS of globus pallidus internus	No improvement in mood symptoms in dystonia patients treated with DBS.
Stavrinou et al. [79]	2019	Greece	*n* = 10	Prospective	DBS of GPi	No improvement in psychiatric symptoms, but improvement in phonemic verbal fluency, which was retained 12 months post-operatively.
Pillon et al. [80]	2006	France	*n* = 22	Prospective	Bilateral pallidal DBS	Improvements in executive function and memory. No improvement in psychiatric symptoms.
Hao et al. [81]	2023	China	*n* = 30	Prospective	Bilateral subthalamic nucleus(STN) DBS	No improvement in cognitive function or sleep quality after 3 years of stimulation.
Liu et al. [82]	2021	China	*n* = 42	Prospective	GPi or STN DBS	No improvements in psychiatric symptoms or sleep quality at 12 months.
Wadon et al. [83]	2021	UK	*n* = 20	Randomised non-blinded trial	8-week internet-based cognitive behavioural (iCBT) therapy vs. standard treatment	No statistically significant difference in anxiety or depression, although trends towards improvement at 3 months in iCBT group
van den Dool et al. [86]	2019	The Netherlands	*n* = 72	Single-blinded RCT	Specialised physical therapy vs. regular physical therapy programme	Both groups showed significant improvements in anxiety and depression, with no between-group difference observed.

RCT = randomised controlled trial; BoNT = Botulinum Toxin; *n* = number of patients.

## Data Availability

No new data were created or analysed in this study. Data sharing is not applicable to this article.
